# Hemoperfusion with Seraph-100 in septic patients removes pathogens and improves clinical outcomes

**DOI:** 10.1038/s41598-025-01280-z

**Published:** 2025-05-21

**Authors:** Antonio Lacquaniti, Antonella Smeriglio, Fabrizio Ceresa, Susanna Campo, Daniele Caruso, Giuseppe Falliti, Erminia La Camera, Francesco Patané, Domenico Trombetta, Paolo Monardo

**Affiliations:** 1 Nephrology and Dialysis Unit, Papardo Hospital, 98158 Messina, Italy; 2 Department of Chemical, Biological, Pharmaceutical and Environmental Sciences, University of Messina, 98166 Messina, Italy; 3 Cardiac Surgery Unit, Papardo Hospital, Messina, Italy; 4 Clinical Pathology Unit, Papardo Hospital, Messina, Italy

**Keywords:** Seraph-100, Hemoperfusion, Sepsis, AKI, ICU stay, Microbiology, Nephrology, Renal replacement therapy

## Abstract

Hemoperfusion (HP) represents a treatment option for sepsis. This study evaluated Seraph-100 in septic patients admitted to the intensive care unit (ICU) after cardiac surgery due to infective endocarditis (IE). Thirteen septic patients were enrolled and treated by Seraph-100 hemoperfusion. Fiftenne patients, not treated by HP, represented a control group. Pathogens were assessed before (T0) and after 4 h of HP treatment (T4). The difference between the two- quantification cycle (Cq) values (T0 and T4), namely ∆Cq at the polymerase chain reaction, was a surrogate marker of pathogen removal. The bacterial load decreased after Seraph-100 HP, with a mean ∆Cq values of 4.6 ± 2.4, as corroborated by conventional haemoculture’s results. Field Emission Scanning Electron Microscopy analyses confirm the Seraph’ adsorptive properties. Procalcitonin, C reactive protein and lactates significantly decreased, with a reduced ICU stay in the Seraph group. After HP, only 15% of patients had AKI requiring renal replacement therapy (RRT), significantly lower than that found in the control group (40%). The Seraph-100 HP induces a decrease of vasopressor doses, a hemodynamic stability and a reduction of AKI and RRT, improving the clinical course, reflected as a reduced ICU stay.

**Supplementary Information:**

The online version contains supplementary material available at 10.1038/s41598-025-01280-z.

## Introduction

Sepsis, inducing 11 million of deaths and accounting for up to 20% of all global deaths, is a worldwide public health concern^1^.

These tragic epidemiological data are attenuated by improving antibiotic therapies and diagnostic strategies, precociously detecting and treating the imbalance between pro- and anti-inflammatory cytokines, and the immune system dysfunction characterizing the septic patient.

Despite these progresses, the heterogeneity of the phenotype that defines sepsis is a not negligible issue for clinical trials, failing to indicate strong recommendations, independently from the sample size of enrolled patients. An early diagnosis induces an early administration of appropriate antibiotics, within the first hour of sepsis detection, concurrently with organ support, representing one of the most effective interventions to decrease mortality, as advised by the Surviving Sepsis Campaign guidelines^2,3^. This aspect has been strengthened also by several publications on sepsis and septic shock, which highlighted a direct correlation between delayed antibiotic administration and adverse outcomes^4,5^. This consideration justifies the comparison between therapeutic strategies applied to the sepsis, acute myocardial infarction, or stroke management, in which the chronological approach is the key for the patient prognosis.

However, sepsis is an inflammatory disease marked by immune dysfunction and the monotherapy is not sufficient to manage it, including the precocious antibiotic treatment, failing to block particular pathways or disimmune processes^6^.

Furthermore, viral forms of sepsis do not benefit from antibiotic therapy unless a secondary bacterial infection occurs, as assessed during the severe acute respiratory syndrome coronavirus-2 pandemic, highlighting that different strategies for managing all forms of sepsis are needed, ranging from managing inflammation, modulating immune responses, and/or modulating metabolism^7^.

Moreover, the effects of antibiotics to counteract sepsis are compromised by the multidrug resistance (MDR), as revealed in pathogens categorized under the well-known abbreviation “ESKAPE”, including *Enterococcus faecium, Staphylococcus aureus, Klebsiella pneumonia, Acinetobacter baumannii, Enterobacter spp., and Pseudomonas aeruginosa*^8^.

In the clinical practice, empirical antimicrobial treatment for septic patients, which anticipates a precise diagnostic confirmation, or their excessive administration in terms of doses and time, contributes to bacteria MDR including several patient-specific factors, such as comorbidities, immunosuppression, and advanced age^9^.

Another overlooked aspect is that the administration of antibiotics even before the sepsis occurs or worsens, or before blood culturing, hinder the diagnosis of pathogens^10,11^.

One meta-analysis, enrolling more than twenty-two thousand septic patients, revealed that positive blood cultures accounted for 40%^12^. Multiple factors are responsible for this false negative result, including the high frequency of blood cultures performed in non-infectious inflammatory conditions^13^. Moreover, conventional blood cultures can only detect live bacteria, not revealing pathogen associated molecular patterns (PAMPs), exogenous microbial products, such as lipopolysaccharide and nucleic acids, which trigger and enhance inflammatory responses^14^.

Conversely, molecular rapid diagnostic tests, including the polymerase chain reaction (PCR) and the matrix-assisted laser desorption/ionization-time of flight (MALDI-TOF) mass spectrometry has reduced the organism identification time, optimizing antimicrobial therapy, and subsequently improving clinical outcomes and mortality rates^15–17^. However, the main limitation of these methods is the potential false positive result due to the detection of extracellular nucleic acids or nucleic acids released from dead bacteria^18^.

Indeed, routine blood culturing takes 2–3 days to identify live bacteria and even more time to test for antibiotic sensitivity, representing a negative prognostic factor, delaying the start of treatment, the precision and the personalization of the therapy, and increasing the morbidity and the mortality rate^19^. Besides these issues related to the appropriateness of the antibiotic prescription, the poor prognosis of these patients is also complicated by sepsis per se, with the natural and acquired immune dysfunction inducing an imbalance between pro- and anti-inflammatory mechanisms, with a final septic phenotype of systemic inflammation, hyperimmune activation, and immunosuppression^20^.

Several biomarkers have been proposed as tools to distinguish sepsis from other non-inflammatory syndromes, such as the procalcitonin (PCT), or the Mid-regional pro-adrenomedullin (ADM), a peptide-like procalcitonin belonging to the calcitonin peptide family, to obtain early the differentiation between inflammation and infection processes^21,22^.

Distinguishing between bacterial and viral aetiologies is another key point to treat these patients.

A potential approach is focusing on the host immune response through the analysis of blood tumor necrosis factor-related apoptosis-inducing ligand (TRAIL), interferon-γ-induced protein-10 (IP10), and C-reactive protein (CRP)^23^.

The MeMed BV® (BV) test distinguishes between bacterial and viral infections analysing the circulating levels of these three immune proteins, changing their expression differentially in response to a bacterial or viral acute infection^24^.

The last step, after a precocious diagnosis of infection, is to manage and treat it, through a multidisciplinary approach, escalating the intensity of treatment according to the progression of the disease. In this context, recently, adsorptive extracorporeal therapies are increasingly applied to the treatment of inflammatory states and sepsis^25^.

Whereas some haemofilters, applied or not to renal replacement therapies, could reduce the cytokine storm or the endotoxin levels, characterizing the septic process, literature data are emerging about the role and the efficacy of the Seraph-100 haemofilter in removing pathogens from the bloodstream through the binding with the immobilized heparin, miming the cell surface heparan sulfate interaction^26^.

The key point related to this filter is the following: apply it early, suggesting that “the sooner, the better” is the best condition to prevent a massive cytokine release with consequent inflammation and immune dysfunction.

Moreover, Seraph-100 does not influence the clearance of several antibiotics with consequent positive clinical implications, avoiding sub-therapeutic antibiotic levels, which negatively influence the sepsis treatment and overall survival^27^.

According to these assumptions, this study evaluated for the first time the role of Seraph-100 in septic patients admitted to the intensive care unit (ICU) after cardiac surgery due to infective endocarditis (IE), which carries a disproportionately increased risk for post-operative sepsis due to the bacteria dissemination into the bloodstream from the infected valves^28^.

The effects of this filter were analysed both from a clinical and laboratory point of view, analysing the blood samples before and after treatment as well as sectioning the Seraph-100 filter and analysing it by Field Emission Scanning Electron Microscopy (FE-SEM).

## Patients and methods

### Study design

This observational prospective cohort single-center study was conducted at the ICU of the Papardo Hospital, Messina, Italy, enrolling thirteen patients admitted to the ICU with a sepsis diagnosis.

The hemoperfusion session was carried out using PrisMax® (Baxter Healthcare Corporation, Chicago, IL, USA) and Multifiltrate (Fresenius Medical Care, Deutschland) systems.

Adult patients with bacteraemia or candidemia secondary to IE, who underwent cardiac surgery, admitted to the ICU and developed a septic process, were set as inclusion criteria.

The surgical approach was established according to the patients’ clinical features such as new onset of heart failure, severe valve regurgitation, echocardiographic signs of haemodynamic compromise, large vegetations, correlation between infection and resistant or very virulent organisms.

Moreover, only patients who received at least one vasoactive drug (dopamine, dobutamine, epinephrine, norepinephrine, vasopressin, or milrinone) during the early stage of sepsis after the admission to ICU, were included.

**Fig. 1:**
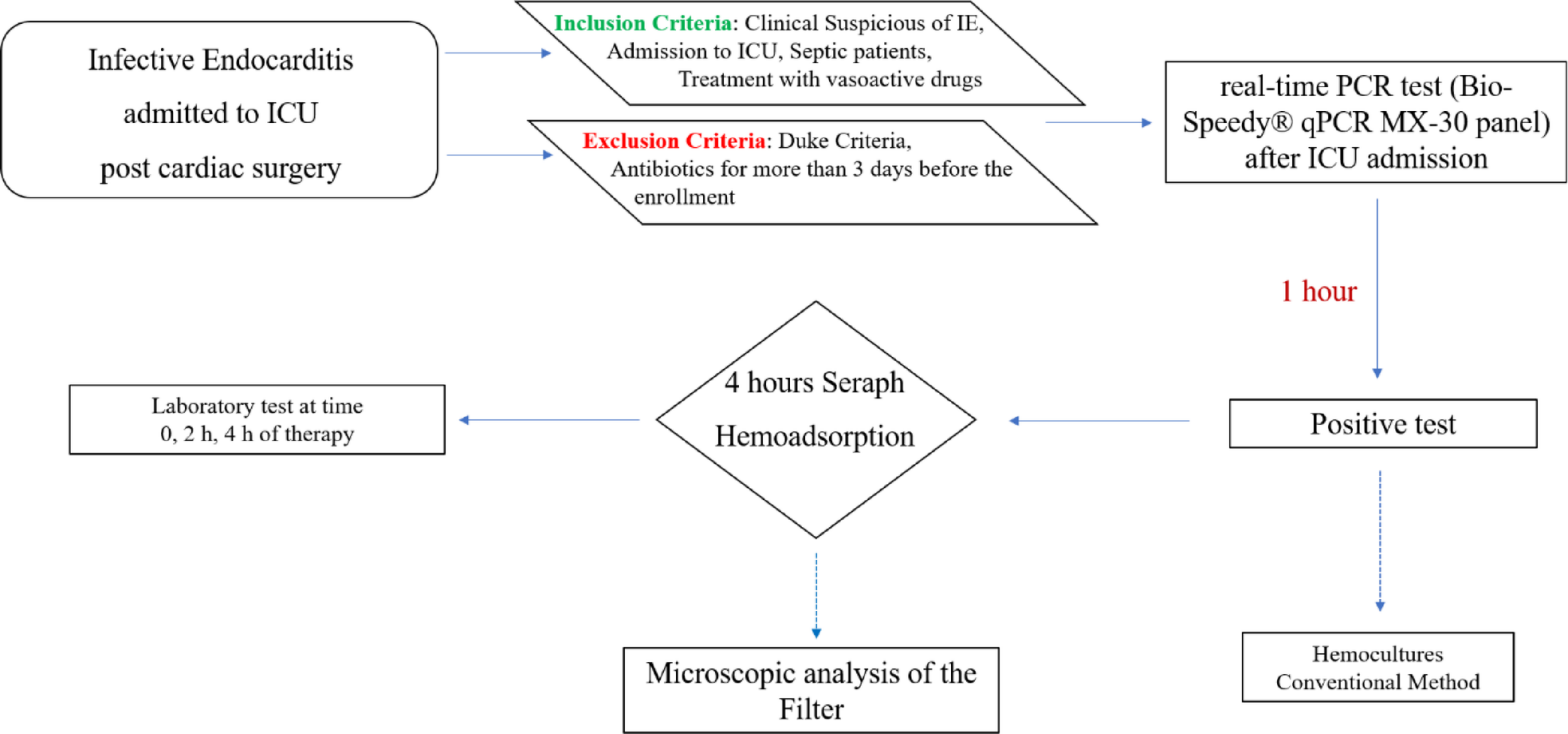
Enrolment plan with inclusion and exclusion criteria. *Abbreviations*: ICU: intensive care Unit; IE: infective endocarditis; PCR: polymerase chain reaction.

Conversely, patients who were classified as “rejected” for infective endocarditis, according to the Modified Duke Criteria^29,30^, or treated by intravenous antibiotics for more than 3 days before the enrolment, were excluded. Figure 1 summarizes the enrolment plan.

Fifteen septic patients admitted to ICU after cardiac surgery due to IE, not treated by hemoperfusion, were retrospectively enrolled from January 1, 2023, to December 31, 2014 as control group. The following information was collected from patients’ electronic medical records: demographic data, comorbidities, microbiological data and transthoracic and trans-oesophageal echocardiographic data—assessing the diagnosis and the severity of the valve endocarditis. (supplementary Figs. 1S-3S and videos 1S-2S).

Data about the inflammatory and infective status, such as CRP and PCT, were assessed in all patients.

The study, complied with the Declaration of Helsinki, was approved by the local Human Research Ethics Committee of the University of Messina, Italy, (protocol number 41–24).

### Definitions

The sepsis diagnosis was based on the Sepsis-3 criteria: documented infection (infection signs and antibiotic treatment for more than 48 h) plus an increase in acute sequential organ failure assessment (SOFA) score of 2 points or more^31^.

The suspected IE was defined by the presence of potential risk factors, such as mitral valve prolapse or degenerative valve disease with reflux on an echocardiogram, valve prosthesis, history of previous infective endocarditis, associated with severe fever (> 37.8 °C), or clinical suspicion of systemic emboli or acute heart failure due to valve dysfunction.

AKI, according to the Kidney Disease Improving Global Outcomes (KDIGO) criteria, was diagnosed in cases of serum creatinine (sCr) level increase by 0.3 mg/dl within 48 h or known or presumed sCr increase until 1.5 times the baseline value within the preceding 7 days, or a urine output of less than 0.5 mL/kg/h for 6 h^32^.

The vasoactive-inotropic score (VIS) was calculated within the first 24 h in the ICU, and every day, until the vasopressors interruption. For the VIS calculation, the following Gaies’ formula^33^ was used:

$$\begin{aligned} C_{f} & = - 2.258 - 0.0172\alpha + 0.00166\text{Re} - 0.0712\varphi _{{{\text{thnf}}}} \\ & \quad - 0.00402{\text{Ha}} + 2.025\tau + 0.000037{\text{Ha}} \times {\text{Ha}} + 0.002761\alpha \times \text{Re} + 0.02337\alpha \times \varphi _{{{\text{thnf}}}} \\ & \quad - 0.000102\alpha *{\text{Ha}} - 0.000005\text{Re} \times {\text{Ha}} - 0.000134\varphi _{{{\text{thnf}}}} \times {\text{Ha}} \\ \end{aligned}$$

### Clinical, biochemical and microbiological tests

Hemodynamic factors, such as mean arterial pressure (MAP) and ventilation parameters, as well as all data related to vasoactive drugs, their dosage, and interruption time, have been recorded.

Biochemical parameters, identifying septic patients with intrinsic prognostic information, such as lactate levels, pH and bicarbonates, CRP, PCT, ADM, and interleukin (IL)-6, were dosed before (T0), after 2 h (T2) and at the end of hemoperfusion treatment (4 h, T4). Furthermore, the same markers were evaluated after 24 and 48 h.

All these measurements were performed in the same laboratory. IL-6 and ADM levels were measured in serum using ELISA (R&D System, Minneapolis, MN, USA and B·R·A·H·M·S KRYPTOR, Hennigsdorf, Germany respectively), whereas CRP and PCT levels were measured using a high-sensitivity immunoturbidymetric method (Modular PPE, Roche Diagnostics GmBH, Mannheim, Germany).

The infectivologists established the antimicrobial therapy according to the current guidelines and local clinical practice, within 1 h, in patients for whom sepsis was highly suspected, or within 3 h, in patients without shock signs and with a low-to-moderate risk of sepsis^34^.

The MeMed BV® (BV) test (Tirat Carmel Park High-Tech North, Israel) was carried out in all patients before the start of the hemoperfusion session, distinguishing between bacterial and viral infections.

In addition to the conventional hemocoltures, real-time quantitative PCR (qPCR) assays targeting specific genomic DNA regions and mRNA transcripts, and providing results in less than 60 min, were performed by the Bio-Speedy® qPCR MX-30 panel (Bioeksen R&D Technologies, Istanbul, Turkey). This kit was able to detect several Gram Positive *(Staphylococcus aureus, Staphylococcus spp, Enterococcus faecium, Enterococcus faecalis, Streptococcus pneumoniae, Streptococcus spp., Listeria monocytogenes)* and Gram Negative *(Pseudomonas aeruginosa, Klebsiella pneumoniae, Acinetobacter baumannii, Haemophilus influenzae, Klebsiella oxytoca, Pseudomonas spp., Enterobacteriaceae, Stenotrophomonas maltophilia, Escherichia coli, Neisseria meningitidis)* bacteria, as well as several fungi *(Candida glabrata, Candida tropicalis, Candida krusei, Candida albicans and Candida parapsilosis)*. Moreover, vancomycin (VanA and VanB), carbapenem (OXA 48, KPC, NDM, VIM, IMP), and methicillin (mecA + C) resistance was also assessed. Data were processed and analysed by the Sigmoida Software Version 7.0.7 (Bioeksen R&D Technologies, Istanbul, Turkey). The pathogen load into the bloodstream was assessed at T0 and T4, calculating the quantification cycle (Cq) values, defined as the fractional number of cycles needed to reach the fluorescence quantification threshold^35^.

The difference between the two Cq values, defined as ∆Cq, accepted as the exponent of the simplified equation to calculate the gene expression ratio, was used as a surrogate marker to reveal the pathogen load decrease from the bloodstream^36,37^.

Furthermore, to establish the mean capacity of the adsorber filter to remove the pathogens, the arithmetic means of the Cq and ∆Cq values for each patient was calculated, according to an accepted equation, justifying the use of the mean Cq of replicate measurements in calculations of gene expression ratios or fold difference^38,39^.

Finally, the Sigmoida software automatically assigned a threshold level according to the shape of the amplification curves obtained by the Magnetic Induction Cycler (Mic) (Bio Molecular System—BMS) Real-Time PCR system. All sigmoidal curves above the threshold level were recorded as positive. On the contrary, non-sigmoidal curves were recorded as negative according to the limit of detection (LOD), that is the lowest detectable concentration of target organisms at which 95% or greater positive detections among all replicates tested were recorded.

As control, conventional haemocultures of properly diluted blood samples, were also carried out in general culture media such as Tryptic Soy agar (TSA) and blood agar (BA) before (T0) and at the end of hemoperfusion session (T4), incubating for 48 h at 37 °C and expressing the results as colony-forming units (CFU). MALDI-TOF-MS analysis (Maldi Biotyper Sirius, Bruker, Milan, Italy), as well as real-time PCR test, through the Bio-Speedy® qPCR MX-30 panel, were carried out to identify the pathogen strains in plate cultures.

### Seraph analysis

To evaluate the pathogen-binding capacity of the filter and its possible selectivity for a specific bacterial strain, the filter was analyzed by a blinded microbiologist at the end of the hemoperfusion session. Specifically, the filter, cut under sterile conditions, was used to prepare suitable suspensions (5 mg/mL) that were seeded (50 µL) in the above general culture media TSA and BA.

Cultures were incubated for 48 h at 37 °C. MALDI-TOF-MS analysis (Maldi Biotyper Sirius, Bruker, Milan, Italy), as well as real-time PCR test, through the Bio-Speedy ® qPCR MX-30 panel, were carried out to identify the pathogen strains in plate cultures.

Finally, to verify that the microorganisms had effectively adhered to the surface of the filter stationary phase, samples of the filter’ sections content were also investigated through FE-SEM analysis with a ZEISS SUPRA 40 VP (Carl Zeiss S.p.A., Milan, Italy) at the acceleration voltage of 20 kV. At this purpose, different filter stationary phase samples (1 mg/each, n = 10) were fixed with 2% glutaraldehyde and processed according to Seffer^40^.

### Statistical analysis

Statistical analyses were performed through NCSS for Windows (version 4.0), Med-Calc software (version 20.115; MedCalc Software Acacialaan, Ostend, Belgium), and GraphPad Prism (version 9.4.1; GraphPad Software, Inc., San Diego, CA, USA) package.

Data were presented as mean ± SD, median (range), or percentage frequency, as appropriate.

A paired two-sided Student T-test was used to determine differences between infective and inflammatory markers in the Seraph group, whereas unpaired t-test was used to evaluate the differences between the two enrolled groups. Pearson or Spearman correlation coefficients were used to test the correlations between ∆Cq, as surrogate marker of decrease in bloodstream bacteria load and other inflammatory and infective biomarkers. Before correlations were tested, all non-normally distributed values were log-transformed to better approximate normal distributions. All results were considered statistically significant for *p* < 0.05.

## Results

### Baseline characteristics of the study population

Table 1 summarizes the main baseline characteristics of the cohort consisting of 28 septic patients.

**Table 1:** Baseline demographic, clinical, and laboratory data of the investigated population.

	Seraph group (n: 13)	No-Seraph group (n: 15)*
Age, mean ± SD	45.7 ± 9.2	49.2 ± 7.2
Male, n (%)	6 (46)	8 (53)
Female, n (%)	7 (54)	7 (47)
Blood hypertension n (%)	10 (77)	12 (80)
Hyperlipidemia n (%)	8 (61)	10 (66)
Smoking n (%)	6 (46)	5 (33)
Diabetes n (%)	9 (69)	8 (53)
SOFA, median (IQR)	3.8 (3.3–5.4)	4.1 (3.7–5.3)
Aortic cross-clamp time, min	101 ± 37	97 ± 31
Cardiopulmonary bypass time, min	112 ± 58	106 ± 48
Pathogens into the blood stream		
*Staphylococcus aureus, n (%)*	3 (23)	4 (26)
*Enterococcus spp., n (%)*	5 (38)	6 (40)
*Enterobacteriacee spp., n(%)*	1 (8)	1 (7)
*Pseudomonas spp., n (%)*	1 (8)	1 (7)
*Streptococcus spp., n (%)*	2 (15)	3 (20)
*Candida parapsilosis, n (%)*	1 (8)	0
Clinical and laboratory parameters		
Lactate, mmol/L	6.6 ± 2.1	7.1 ± 3.3
VIS, points	36 [21–49]	33 [18–42]
MAP, mmHg	56 ± 8.1	57.4 ± 5.8
PCT, ng/mL	43.5 ± 23.2	39.7 ± 13.5
CRP, mg/L	34.7 ± 13.9	32.2 ± 10.3
ADM, nmol/L	7.5 ± 2.7	–
IL 6, pg/mL	419.1 ± 112.9	–

The two groups have been well matched for demographic, comorbidities and intra-operative characteristics. The main comorbidities, represented by hypertension (77%), dyslipidemia (61%), diabetes (69%), and smoke (45%) in the Seraph group, were well matched with the control group. Before surgery, all patients underwent transoesophageal echocardiography. In the Seraph group, valve vegetations were detected in 7 patients, abscess in 3 patients, whereas 3 patients had valve perforation. The most common infected valve was the aortic valve (9 patients), followed by the mitral valve (2 patients). As endocarditis complications, heart failure NYHA III/IV was detected in 3 patients. Similar conditions were also observed in the control group, in which vegetations were assessed in 8 patients. The aortic valve was also in this case the most affected.

The mean aortic cross-clamp time was 101 ± 37 min, whereas the mean value of the cardiopulmonary bypass time was 112 ± 58 min. These times of the surgical procedure were not statistically different in the control group.

All patients were transferred to the ICU after the surgery. The mean ICU stay in the Seraph group was shorter than in the control group (10.8 ± 5.3 days; median 8 days; IQR: 4–11 days vs. 14.6 ± 2.7; median 12 days; IQR: 6–17 days; *p*: 0.02).

No patients suffered from chronic kidney disease before the surgical treatment. In the Seraph group 2 patients (15%) had AKI requiring renal replacement therapy (RRT), whereas this complication was more pronounced in the control group, where 6 patients (40%) needed RRT.

### Seraph group: microbiological results and clinical effects

The microorganisms isolated by the real-time PCR test through the Bio-Speedy® qPCR MX-30 panel and analysed by MALDI-TOF-MS system were *Staphylococcus aureus, Enterococcus spp., Pseudomonas spp., and Streptococcus spp.,* whereas in one patient a candidemia, due to *Candida parapsilosis*, was revealed. These pathogens were responsible for the infection inducing the IE. Penicillin, ampicillin, gentamicin, streptomycin, ceftriaxone, ampicillin/sulbactam, vancomycin, daptomycin, ceftaroline, linezolid, amphotericin B were the antibiotics administered during the ICU stay.

No concomitant infections were revealed, identifying one isolated pathogen per patient.

MEMED test revealed a specificity and sensibility around the 100%, revealing precisely bacterial infection in all patients, according to its levels ranging from 85% to 98%. Moreover, its value < 30, suspicious for non-bacterial infection, was assessed in the patient with candidemia.

Conventional haemocultures, followed by MALDI-TOF-MS analysis confirmed these data in 6 patients (46%), whereas, in the remaining, negative results were recorded.

All these patients belong to the Seraph group and no adverse events related to the device or procedure were reported.

**Fig. 2:**
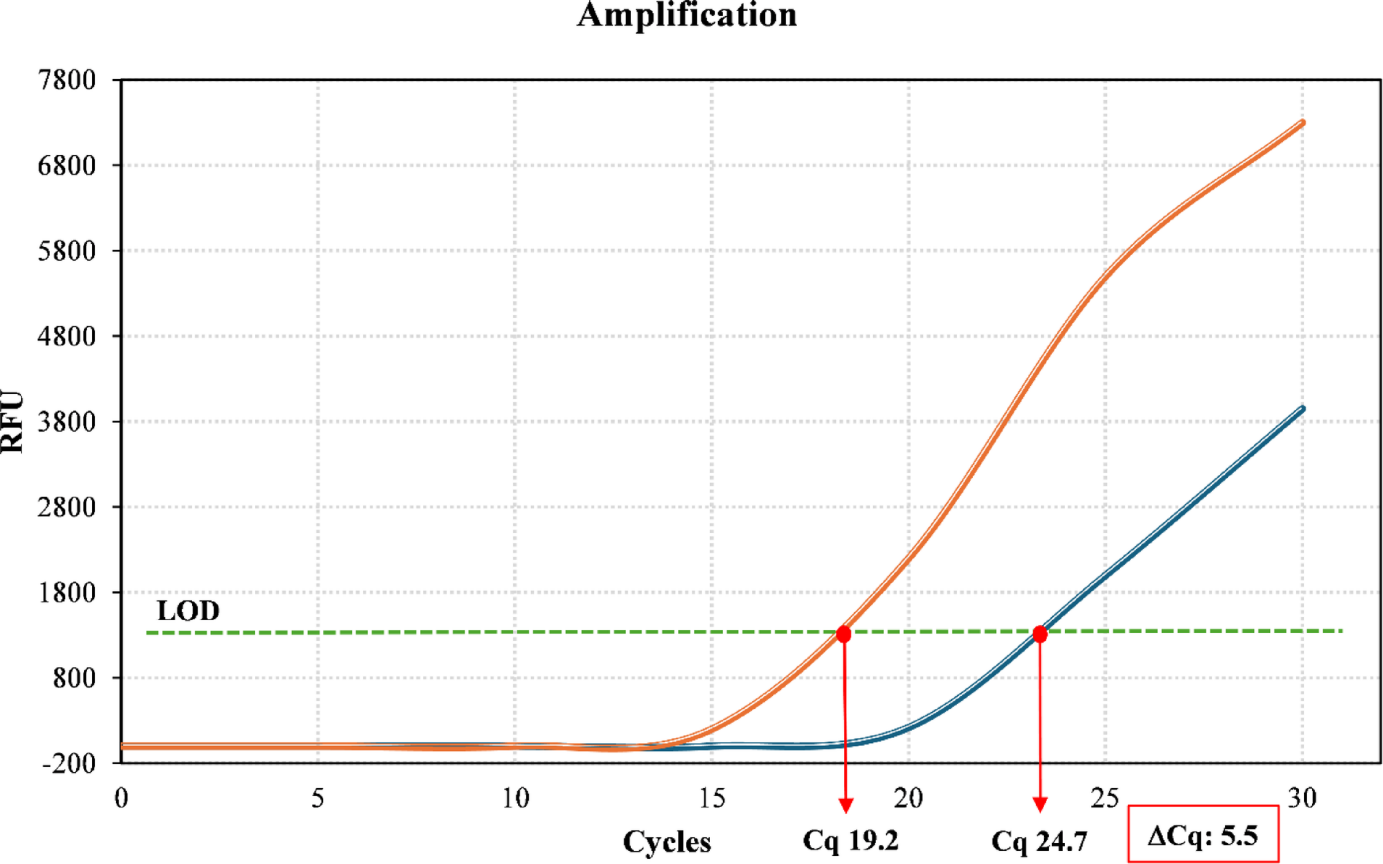
qPCR test performed in a Seraph-treated septic patient. The bloodstream pathogen load was assessed before (orange curve) and at the end (blu curve) of the hemoperfusion session (4 h). The Cq values, defined as the fractional number of cycles that were needed for the RFU to reach the quantification threshold, were inversely related to the pathogen burden. The difference of the two Cq values, defined as ∆Cq, assessed at the beginning and at the end of the hemoperfusion session, is a surrogate marker of the bloodstream pathogen burden reduction, reflecting the capacity of the adsorber filter to remove the pathogen. *Abbreviations*: Cq: quantification cycle; LOD: limit of detection; RFU: fluorescence; ∆Cq: differences between the two Cq values revealed before and after the Seraph treatment.

By analysing the data obtained by the sigmoidal curves of the PCR assays, a significant decrease in the bacteria load was recorded at the end of the hemoperfusion session (4 h). In particular, a CqT4 decrease was observed in each Seraph-treated patient (CqT0: 23.2 ± 2.5 vs. CqT4: 27.8 ± 1.3; *p* < 0.0001), with a mean ∆Cq value of 4.6 ± 2.4, highlighting a statistically significant decrease of the bloodstream bacteria load Fig. 2.

The efficacy of the filter was confirmed also by analysing data of the patient affected by candidemia, whose load, at the end of the hemoperfusion, was significantly reduced, (CqT0: 24.7 vs. CqT4: 29.1; *p* < 0.0001).

**Fig. 3:**
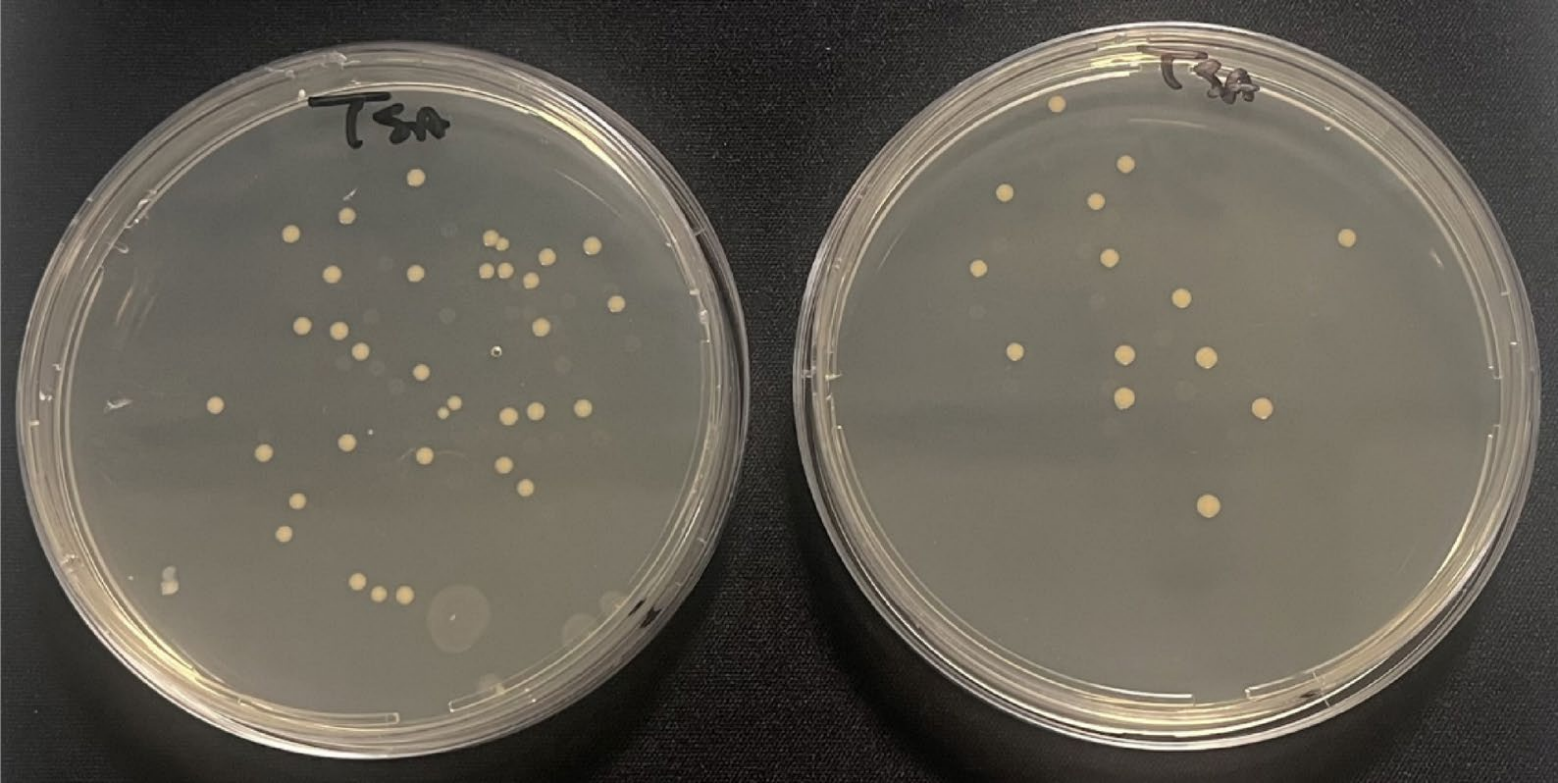
Representative pictures of a blood sample seeded in TSA culture medium plates before (left) and after (right) Seraph hemoperfusion session in which is well visible the Staphilococcus aureus growth. The reduction of the bacterial load is clearly visible from the reduction of the colony-forming units (CFU).

The bloodstream pathogen load was also analysed by conventional haemocultures at the beginning and at the end of the treatment. Only 46% of patients had positive cultures, but the MALDI-TOF-MS analysis of them confirmed what was previously detected by qPCR data, detecting *Staphylococcus aureus*, *Escherichia coli*, *Pseudomonas aeruginosa*, and *Candida parapsilosis*. The CFU in culture media decreased significantly between the beginning (T0) and the end of treatment (T4), corroborating the detected ∆Cq values, with a mean pathogen growth percentage decrease of 46.7 ± 11.2% Fig. 3.

However, all patients maintained positive blood cultures after the Seraph treatment.

**Fig. 4:**
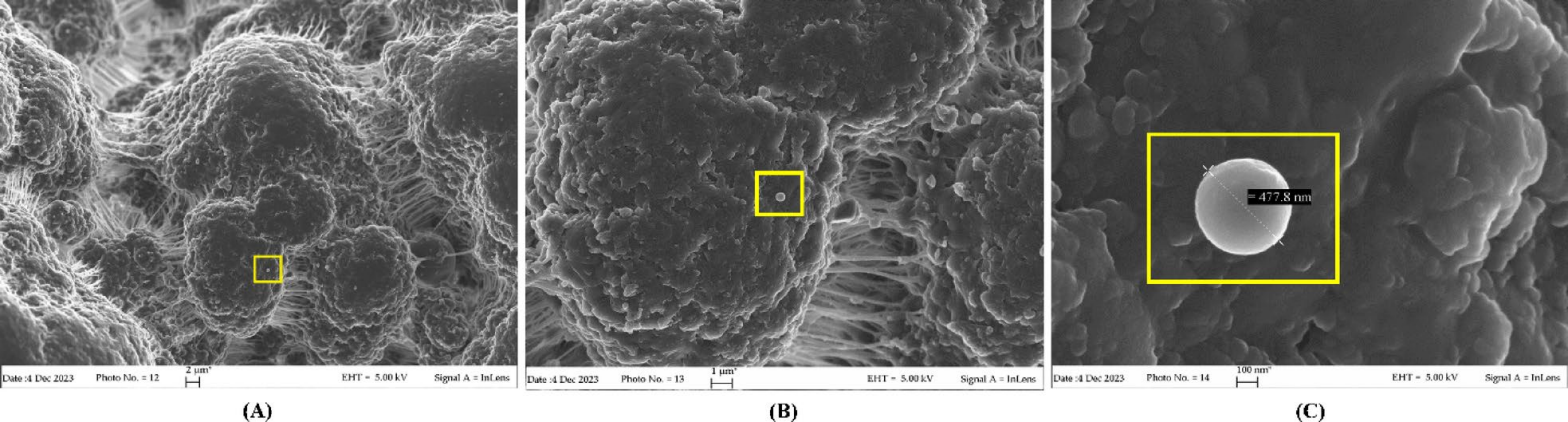
Representative FE-SEM pictures, at different magnification (scale bar 2 µm, 1 µm and 0.1 µm for A, B and C respectively), of the Seraph-100 stationary phase at the end of the hemoperfusion session (4 h), in which is evident the presence of a bacterium comparable in shape and size with Staphilococcus aureus identified by MALDI-TOF analysis.

The capacity of the Seraph filter to adsorb microorganisms was demonstrated not only by the microbial decay curves recorded at the end of the hemoperfusion session and conventional haemocoltures, but also by the analysis of the filter stationary phase at the end of the hemoperfusion session. Indeed, FE-SEM analysis highlighted the presence of microorganisms compatible in shape and size with those recorded by MALDI-TOF-MS and Real-time PCR analyses Fig. 4.

From a clinical point of view, all patients were monitored during the ICU stay, with all parameters employed in the common clinical practice.

**Fig. 5:**
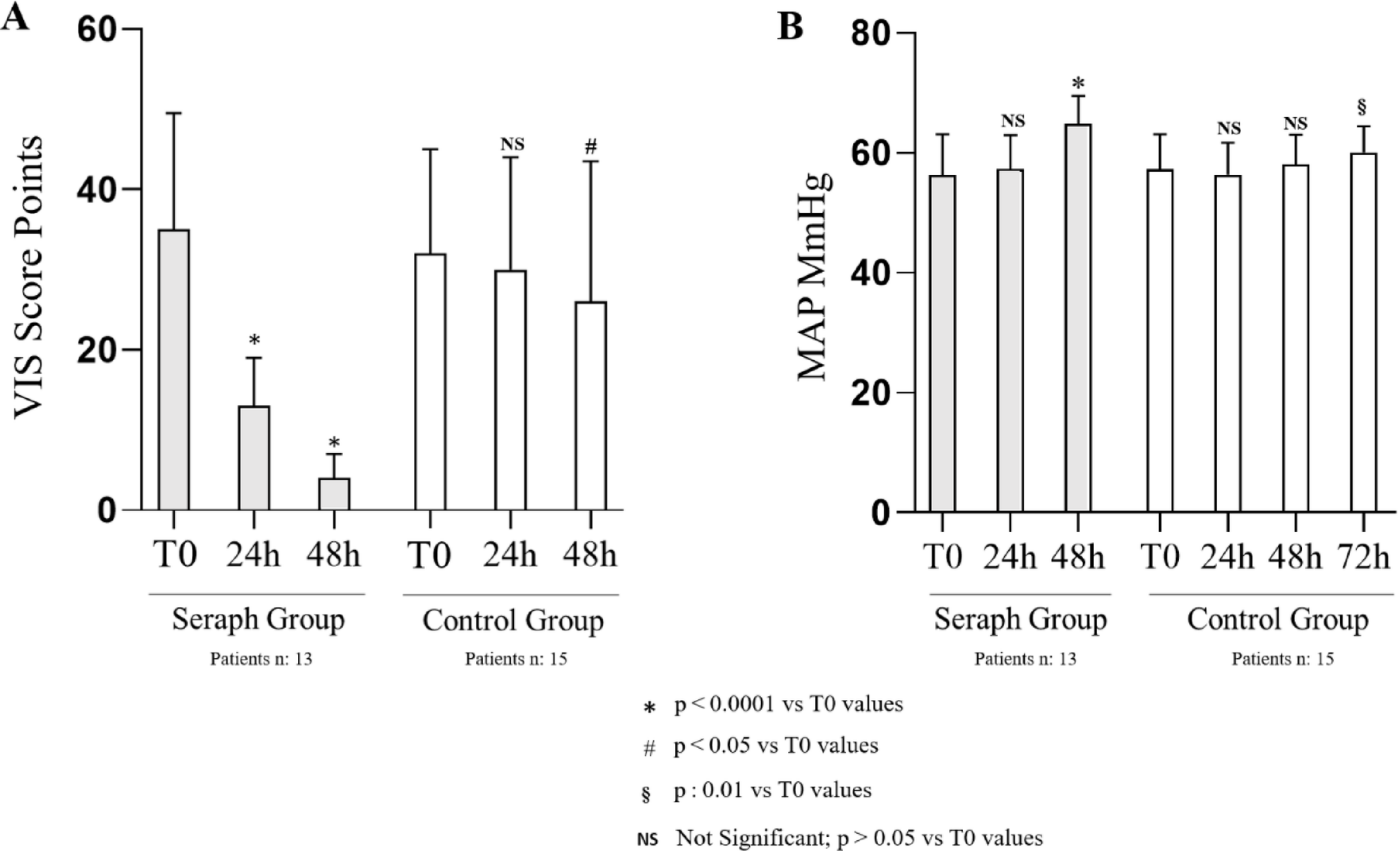
VIS Score and MAP values in the Seraph and Control groups during the first 72 h of ICU stay. *Abbreviations*: VIS: vasoactive-inotropic score; MAP: mean arterial pressure;

During the early stage of sepsis after the admission to ICU, vasoactive drugs were administered in all patients treated by hemoperfusion. In particular, VIS decreased significantly after 24 h (13 [5–18] points, *p* < 0.0001) and 48 h (4 [1–7] points, *p* < 0.0001) in comparison with data recorded at the beginning of the Seraph treatment (36 [21–49] points) Fig. 5A.

In seven patients, the vasoactive drugs were suspended after 48 h from the hemoperfusion session. After 24 h, no statistically significant differences were observed in MAP (T0: 56 ± 6.8 mmHg vs. T24: 57.3 ± 5.6 mmHg; *p*:0.07), whereas its increase has been recorded after 48 h in the Seraph Group (T0: 56 ± 6.8 mmHg vs. 64.7 ± 4.9 mmHg; *p* < 0.0001). Conversely, a significant improvement in MAP values was recorded after 72 h with respect to the ICU admission Fig. 5B.

**Fig. 6:**
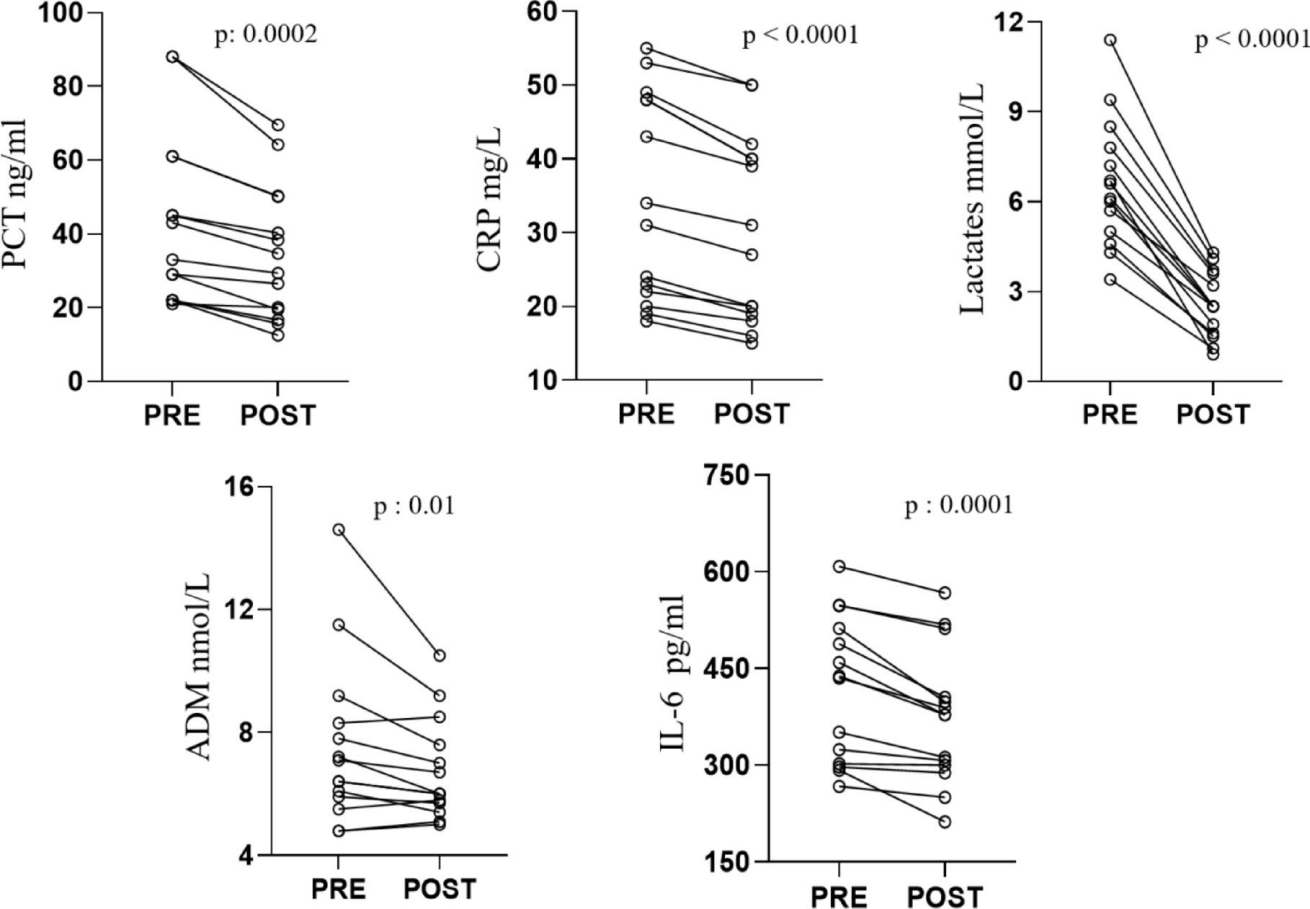
Variations of infective and inflammatory biomarker levels pre and post Seraph-100 treatment. *Abbreviations:* PCT: procalcitonin; CRP: C reactive protein: IL: interleukin; ADM: Mid-regional pro-adrenomedullin.

The inflammatory and infective markers were investigated in all patients by analysing their trend during the four hours of the hemoperfusion session as well as after 24 and 48 h. At T2 and T4 samplings, the treatment did not induce any decrease of marker levels, if compared with the baseline values assessed at the beginning of the hemoperfusion session. Conversely, an initial decrease was observed after 24 h. In particular, PCT (43.5 ± 23.2 ng/mL to 34.8 ± 18.2 ng/mL; *p*: 0.0002) and CRP (34.7 ± 13.9 mg/L to 30.5 ± 12.7 mg/L; *p* < 0.0001) a decrease was recorded 24 h after the end of the treatment. Similarly, ADM and IL-6 levels decreased after 24 h (7.5 ± 2.7 nmol/L to 6.7 ± 1.6 nmol/L; *p*: 0.01 and 419.1 ± 112.9 pg/mL to 372.4 ± 104.3 pg/mL; *p*: 0.0001, respectively). A progressive decrease of the lactate levels was also observed within the 48 h of the end of treatment (from 6.6 ± 2.1 mmol/L to 2.5 ± 1.1 mmol/L; *p* < 0.0001) Fig. 6.

A control group, which well-matched with the Seraph group for all baseline clinical and laboratory parameters, was retrospectively enrolled (Table 1). As above mentioned, the clinical course of these patients was worse, as highlighted by the longer ICU stay and the increased need for RRT. In particular, a slower VIS score decrease was recorded, with statistical differences with respect to the baseline levels only after 48 h from the ICU admission (baseline: (33 [18–42] points); 24 h: (30 [16.5–44] points) *p*: 0.07; 48 h: (26 [14.5–43.5] points) *p*: 0.03). Moreover, the interruption of vasopressors was achieved in 3 patients after 3 days from the ICU admission. A similar trend was assessed for lactates, PCT and CRP, whose levels statistically decreased from the baseline only after 72 h. Similarly, MAP increased slowly, with improved values and statistically significant results recorded only after 72 h from the ICU admission. ADM and IL-6 were not tested in the control group. Moreover, only traditional methods were used to identify the pathogens in the bloodstream, with positive cultures observed in 7 patients (47%), with a mean time of results obtained after 4.7 ± 1.2 days from the blood collection.

### Seraph group: correlates of Cq and ∆Cq

**Fig. 7:**
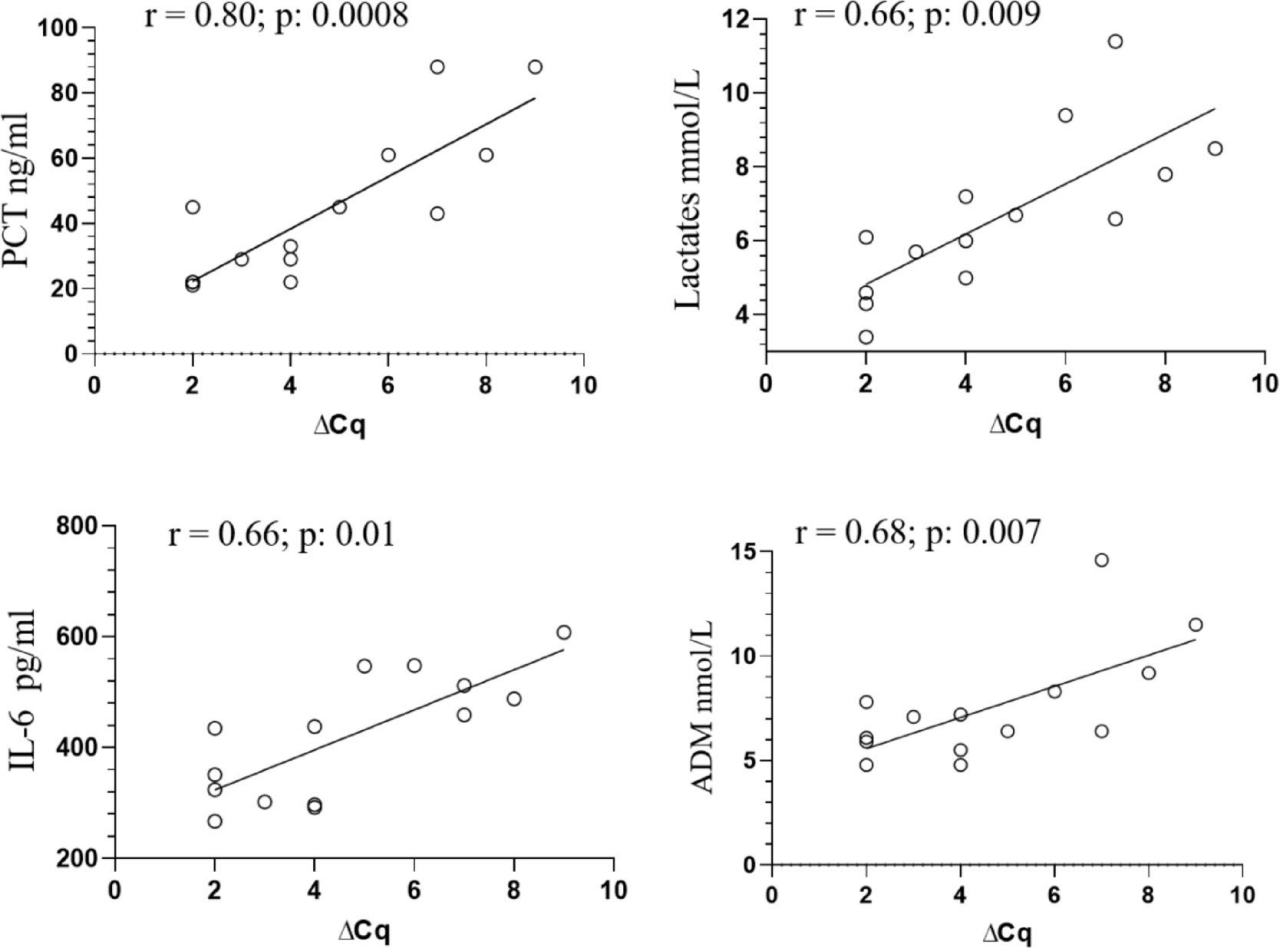
Correlations between ∆Cq and infective and inflammatory biomarkers. *Abbreviations*: PCT: procalcitonin; CRP: C reactive protein: IL: interleukin; ADM: Mid-regional pro-adrenomedullin; ∆Cq: differences between the two Cq values revealed before and after the Seraph-100 treatment.

By univariate analysis, the baseline Cq value was found to be inversely correlated to the ADM (R = − 0.72, *p*: 0.003), IL-6 (R = − 0.74, p: 0.002), lactate levels (R = − 0.78, *p*: 0.0008) and PCT (R = − 0.78, *p*: 0.0008). Considering ∆Cq as surrogate marker revealing the reduction of pathogen load from the bloodstream, its correlation with biomarkers assessed after the end of the treatment was evaluated. ∆Cq was found to be directly correlated to ADM (R = 0.68, *p*: 0.007), IL-6 (R = 0.66, *p*: 0.01), lactate levels (R = 0.66, *p*: 0.009), and PCT (R = 0.80, *p*: 0.0008). Figure 7. provides a graphical summary of these findings.

However, using ∆Cq as dependent variable in a multiple regression model including all previously reported univariate correlates, only the PCT correlation (β = 0.68, *p*: 0.0005) remained significant.

## Discussion

Findings from the present study showed that Seraph-100 hemoperfusion, represents a specific treatment for bacteremic patients who underwent cardiac surgery due to IE, reducing the pathogen load from the bloodstream.

Beyond the innovative nature of this study, which certainly provides important evidence on a possible appropriate use of Seraph in these patients, there are some limitations to highlight. First, the sample size was relatively small, and data derived from a single cohort. Second, the inclusion criteria were based on qPCR data, with potential bias secondary to the method, such as false positive results due to revealed genetic fragments not related to pathogen burden. Moreover, no literature data are available to rule out potential adsorption of DNA or RNA to Seraph, inducing false positive results of qPCR reduction, with the need of further studies to confirm or not this hypothesis. Third, as the study specifically focused mainly on patients with bacterial infection, the results may not be generalizable to a broader, unselected sepsis population, even if in our cohort a patient affected by candidemia was enrolled. Finally, this study does not evaluate the impact of the inflammation and altered hymmune host response in these patients, highlighthing that distinguishing the infection from the inflammation represents a challenge in septic patients after cardiac surgery.

Conversely, among the strengths of this study is the use of a well-defined cohort, consisting entirely of patients with confirmed pathogens in the bloodstream, through bio-molecular analyses, conventional haemocultures and microscopic confirmations. Moreover, the entire study population was well-matched for comorbidities, intraoperative parameters, demographic and laboratory data, to obtain clinical implication attributable to the hemoperfusion and Seraph application.

The immediate effects are, in most of patients, the reduction and sometimes suspension of the vasoactive drugs, the decreased inflammatory and infectious biomarkers levels, and the improvement of hemodynamic instability.

In all Seraph-treated patients, a decrease of the vasoactive drugs dosage within 48 h from the end of the treatment was observed, and in some cases, a complete suspension within two days. In the control group, treated by the well-established strategies recommended by current guidelines, vasopressors were reduced much more slowly, as highlighted by the different trend of the VIS score, an important mortality and morbidity predictor^41–43^.

Moreover, the vasopressors reduction in the early phase of sepsis could avoid the side effects often observed in these patients, independently from the maintenance of blood pressure, such as the severe vasoconstriction altering the microcirculation, arrhythmias, and organ dysfunction^44–46^. Whereas sepsis guidelines suggest the administration of numerous vasoactive agents, the timing of their use or discontinuation is not defined, with the clinical issue of managing positive and negative effects, personalizing the treatment with the main target of maintaining adequate pressure and improving the microcirculatory perfusion.

In this study, it has been demonstrated for the first time that a precocious reduction of the pathogen bloodstream load counteracts the cascade of events leading to hemodynamic alterations,

associated with a reduction of all inflammatory and infective markers, including the lactate levels.

Finally, a progressive MAP improvement, a hemodynamic indicator of appropriate perfusion pressure, representing a widely accepted target for resuscitation and vasopressor titration^47^, was observed.

The target MAP (65 mmHg), recommended by the Surviving Sepsis Campaign guidelines to maintain critical organ perfusion, has been achieved after 48 h from the end of the hemoperfusion session, 24 h before, if compared to the control group, in which the increase of the MAP values was observed only after 72 h from the ICU admission^34^. These data, reflecting an improved hemodynamic condition, explain the better clinical course of the Seraph-treated patients, the reduced ICU stay and a precocious transfer to the clinical ward.

The main strength of this study was the enrolment of a homogeneous cohort of septic patients. This condition is not easily expected in the clinical practice, with microbiological conventional methods which fail to identify pathogens and to give precocious information to clinicians if a positive test occurs. In fact, in the control group, the conventional haemocultures results were received only four days after the sepsis diagnosis, and only in 46% of patients the pathogen was identified starting a personalized antibiotic therapy.

On the contrary, in the Seraph group, microorganisms were isolated and identified within 1 h by a real-time PCR test and MALDI-TOF-MS analysis. Moreover, the suspicious of an infective event was corroborated by the MEMED test, revealing, in our patients, high diagnostic specificity and sensibility. All these techniques could improve the diagnostic processes, in terms of sensibility and the specificity, in the cardiac surgery context, representing the starting point for a personalized approach in IE patients.

Our study, therefore, strengthens literature data suggesting qPCR and MALDI-TOF MS methods as rapid diagnostic techniques for the precocious identification of various pathogens, reducing the time needed for antimicrobial therapy, reducing the hospital stay and improving the survival of septic patients^15,17^.

Furthermore, it is important to underline that, in the Seraph group, only the 46% of patients had positive haemocultures, whereas microbial infections were revealed in all the patients through qPCR analysis. The FE-SEM analysis confirmed the universal pathogens binding capacity of Seraph-100, confirming the low sensitivity of the conventional methods, and corroborating the data obtained by MALDI-TOF–MS and Real-time PCR analyses.

Only an early and accurate identification of pathogens could allow for a personalized therapy, applying the Seraph-100 hemoperfusion, if indicated, to obtain the precocious interruption of the event cascade characterizing the septic processes through the reduction of the bloodstream pathogen load. Interestingly, previous reports have already demonstrated that Seraph-100 did not alter the kinetic of several antibiotics, representing not negligible advantage in terms of maintained efficacy of these drugs in septic patients^27^. Similarly, in this study, no reduction of inflammatory and infective biomarkers were observed during Seraph treatment, revealing similar values between the beginning and after 2 and 4 h after hemoperfusion session. The clinical consequence is that Seraph-100 does not invalidate the diagnostic sensitivity and specificity of these biomarkers. The convective process determines the removal of many sepsis biomarkers, whose reliability as markers in septic patients undergoing RRT is under question, with doubts about their diagnostic and prognostic role in patients treated by RRT^48^.

However, further studies exploring biomarker elimination by Seraph are needed to confirm our hypotheses. The precocious and personalized management of septic patients based on a rapid detection of pathogens in the bloodstream and indication to Seraph treatment, could lead to positive economic effects, as reported in a previous study evaluating other adsorbers. In particular, intraoperative hemoadsorption in IE patients might have relevant economic benefits related to reduced ICU stays if treated with the adsorber filter CytoSorb^49^.

The reduced need for vasopressors, achieving an early hemodynamic stability, as well as the diminished incidence of septic complications, such as AKI and RRT, have induced in the Seraph group a shorter ICU stay, if compared to the control group. However, the economic impact of Seraph was not a target of this study, and further studies evaluating the economic implications are necessary to confirm this hypothesis.

Furthermore, in all these patients, no adverse events related to the device or the procedure were reported during the hemoperfusion session.

In conclusion, this study demonstrates that Seraph hemoperfusion is indicated in septic patients with bacteraemia. This treatment could interrupt the inflammatory and immune system activation, which characterize the early phases of the septic process, reducing the pathogen load, as revealed by bio-molecular methods, conventional haemocultures and FE-SEM analysis. The improvement of clinical course, reflected as ICU stay reduction, has been mediated by the reduction of vasopressors need, hemodynamic stability and avoidance sepsis-related complications, such as AKI and RRT.

Randomized clinical trials are required to confirm our data, establishing the timing of the treatment and supporting and corroborating our hypothesis: “the better the sooner”. Further clinical and laboratory parameters should be selected to achieve the treatment personalization, avoiding interventions that could be applied to unselected patient populations leading to false negative results due to not appropriate indications.

## Electronic supplementary material

Below is the link to the electronic supplementary material.

Supplementary Material 1

Supplementary Material 2

Supplementary Material 3

## Data Availability

The data underlying this article will be shared on reasonable request to the corresponding author.

## Abbreviations

AKI: Acute kidney injury
CRP: C-reactive protein
FE-SEM: Field emission-scanning electron microscopy
GFR: Glomerular filtration rate
IE: Infective endocarditis
ICU: Intensive care unit
IP10: Interferon-γ-induced protein-10
IL: Interleukin
KDIGO: Kidney disease: improving global outcomes
LOD: Limit of detection
MALDI-TOF: Matrix-assisted laser desorption/ionization-time of flight
MAP: Mean arterial pressure
ADM: Mid-regional pro-adrenomedullin
MDR: Multidrug resistance
PCR: Polymerase chain reaction
PCT: Procalcitonin
RRT: Renal replacement therapy
SOFA: Sequential organ failure assessment
sCr: Serum creatinine
TRAIL: Tumor necrosis factor-related apoptosis-inducing ligand
VIS: Vasoactive-inotropic score
