# Supplementary material for: Hemoperfusion with Seraph-100 in septic patients removes pathogens and improves clinical outcomes
Source: Sci Rep. 2025 May 21;15:17626. doi: 10.1038/s41598-025-01280-z (PMC12095501; doi:10.1038/s41598-025-01280-z)
Supplement: Supplementary file 1 — Supplementary Material 1 [file 41598_2025_1280_MOESM1_ESM.docx]

**Supplementary figures and videos**

**Figure 1S** Valve vegetations evaluated at the transthoracic echocardiography

**
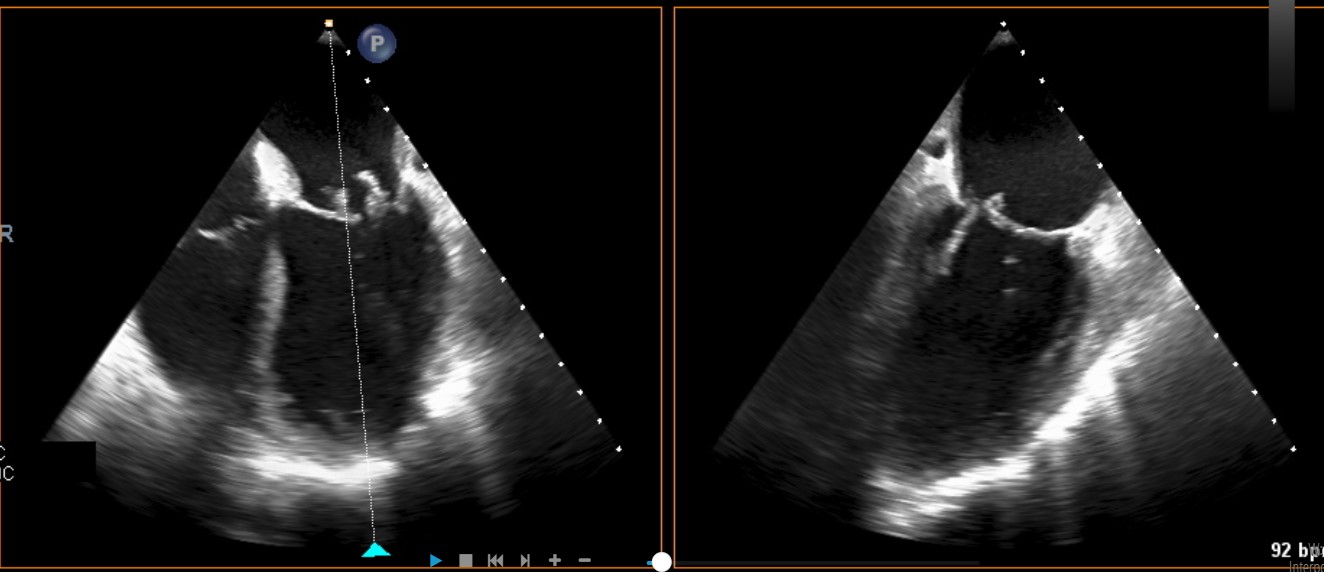
**

**Figure 2S** Valve vegetations evaluated at the transthoracic echocardiography

**
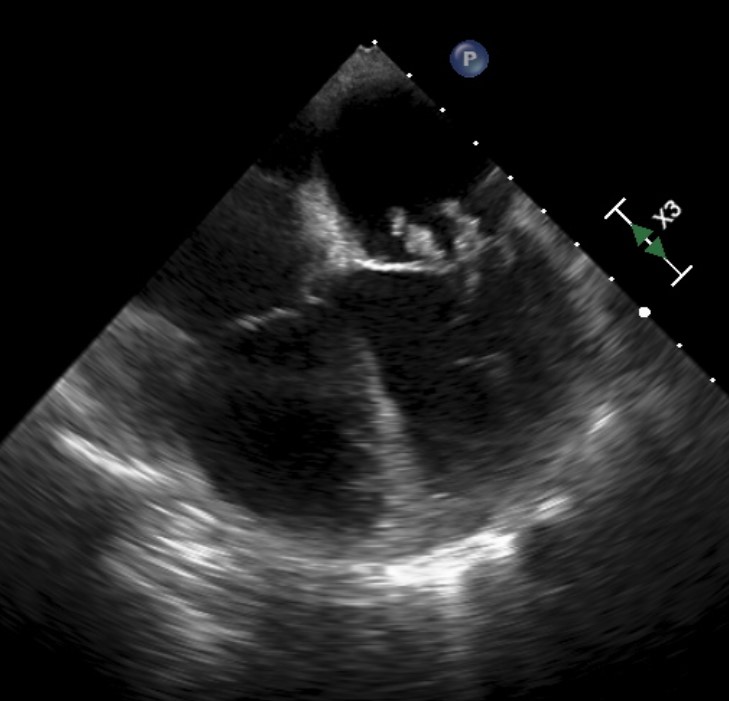
**

**Figure 3S** Valve vegetation evaluated by 3D trans-oesophageal echocardiography

**
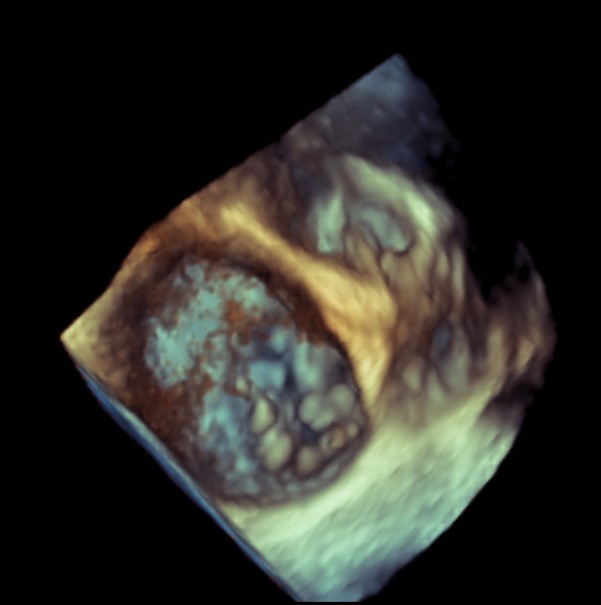
**

**Video 1S** valve vegetations evaluated by transthoracic and 3D trans-oesophageal echocardiography.

**Video 2S** valve vegetations evaluated by transthoracic and 3D trans-oesophageal echocardiography.
